# Exostosis, or Dental Osteoma

**Published:** 1886-08

**Authors:** W. C. Barrett


### EXOSTOSIS, OR DENTAL OSTEOMA.

BY W. C. BARRETT, M. D., D. D. S.

Read at the Twenty-Second Semi-Annual Meeting of the Connecticut Valley Dental Society.

Tumors may be defined as normal tissues abnormally developed, either in position, relative quantity, or structure. Under this definition, dental exostosis must be classed as a benign tumor. It is really a hypertrophy of cementum. Under the stimulus of irritation, many tissues take upon themselves an abnormal growth. The irritation is, perhaps, not sufficiently severe to cause inflammation, but it may stimulate to an increased flow of nutrient material, and this may be so built into tissue as to produce the hypertrophied growth. There may be this condition in two of the tissues of a tooth, the dentine and the cementum. When it takes place in the dentine, besides the thickening of the dentinal walls there may be a deposit within the pulp chamber and connected with the internal pariety that, in some instances, causes intense pain. Or there may be concretions within the substance of the pulp itself, presenting many of the characteristics of dentine, and these are at times the source of great annoyance.

The term Dental Exostosis is, however, commonly confined to an hypertrophied growth of the Cementum. A complete comprehension of this abnormal condition would lead us to an exhaustive study of the character and function of the dental pericementum, as it has been commonly supposed that this membrane is active in the formation of the cementum. I cannot, however, do more than briefly to notice some of the theories concerning its office.

Salter declares the pericemental membrane and the periosteum lining the socket one, a single membrane ministering to the nutrition of the cementum on the one side and the bone on the other. (Dental Pathology and Surgery, p. 14.)

Dr. L. C. Ingersoll, in an exhaustive paper upon this subject, read before the last meeting of the American Dental Association, asserts that it is a double membrane, dipping from the alveolar border into the tooth socket, and reflected back upon the root. (Transactions American Dental Association, p. 126 *et seq.*)

Dr. G. V. Black asserts that it is not a periosteum at all, but is a vascular membrane connecting the tooth with its socket, and has nothing to do with the nourishment of the cementum. (See INDEPENDENT PRACTITIONER for June, 1886, p. 308.) He declares that it is but a fascia, and says that the nutrition of the cementum is wholly due to the osteoblasts which line its surface, but which form no part of the peridental membrane proper. Whatever may be the dental office of this tissue, it is certain that it is highly vascular in its nature, and that it supplies the osteoblasts with nutrition, and that it is so intimately connected with them that it may, for our purpose, be considered as either directly or indirectly supplying the needed pabulum to the cementum of the tooth.

Cementum closely approaches true bone in its structure. There are the canaliculi and the lacunæ of bone, but their arrangement into lamellæ is not as perfect as in true osseous tissue. It is more closely allied to primary bone, or that which has been developed in temporary cartilage, or upon the surface of existing bone. (Dental Surgery; Tomes, p. 424.) The periosteum of bone, under a certain stimulus, will deposit additional layers, or in some cases concretions of true bone. In fractures there is a deposit of what is termed "callus," and this is organized into osseous tissue. We know that in bone it is the office of the osteoblasts to furnish the material of which it is composed, and as cementum so nearly approaches bone in its structure, the process must be analogous.

These osteal cells, or osteoblasts, lie at the immediate surface of the bone, or wherever bone is being developed, whether in cartilage, in membrane, or in periosteum. (Tomes; Dental Surgery, p. 425.) The pericementum, upon its inner surface, is covered with a layer of these cells. They are in immediate contact with the cementum, and under the stress of some undue stimulation they deposit upon it continuous layers, which form that abnornal condition that we call Exostosis.

The structural character of cementum depends largely upon the thickness of the layer. At the cervix of the tooth, where it is very thin, the lacunæ are altogether absent, and the canaliculi are not at all marked. But if sections are made at a little distance from the neck of the tooth, where it is thicker, the canaliculi begin to appear, and further on they are arranged into lacunæ and the canaliculi begin to anastomose, forming a connection between the corpuscles.

True bone is traversed by vascular tracts called Haversian canals. In cementum, as existing in normal thickness, there are no Haversian canals, but in hypertrophied cementum, or where this tissue exists in considerable thickness, the Haversian canals make their appearance, and the resemblance to true bone is almost complete. The deposition of cementum, then, in that condition which we call exostosis, is an analogue of the formation of secondary bone tissue, and must be studied in the same light.

Osteoma, or the formation of osteophytes, has been traced to different kinds of periosteal irritation. They may exist almost anywhere, but hypertrophies of bone tissue are especially common upon the maxillæ. Tomes remarks upon the extraordinary facility with which the jawbones change their shape. (Dental Surgery, p. 430.) Alveolar abscesses erode great hollows in them. Many teeth are extracted, leaving the bone in a very irregular, cavernous condition, yet a little time entirely removes all traces of the injury. The jaw-bones may be expanded by tumors beyond all semblance of their original shape, but removal of the tumor is quickly followed by a restoration of the bone to its normal condition. Large portions of the maxillæ may be excised by surgical interference, but they are quickly restored if the conditions be favorable and the periosteum be not entirely removed. An odontome may expand the bone until but a mere shell remains, but if this be broken down and the tumor removed, it again returns to the shape of a hard bony ridge. It follows, then, that hypertrophies of the jaws being so common, and the cementum of the tooth partaking so closely of the nature of osseous tissue, we may look for frequent exostoses of the teeth. Garretson says (System of Oral Surgery, p. 904), that a thousand instances of hypertrophied cementum are found to that of one of any other osseous tissue, and the teeth are subjected to a thousand times the irritating influences of any other of the bones. In fact, so active is the investing membrane of the tooth-root, and so liable to irritations of various kinds, owing to the constant and often violent use of the teeth and their subjection to great and varied changes, that the cementum is never a fixed quantity, but it may be said to be always varying in amount. It undergoes constant absorption and redisposition. (Salter; Dental Pathology and Surgery, p. 129.)

The abnormal deposition of cementum is not dependent upon the vitality of the tooth pulp. In fact, it seems most excessive in teeth that have long been devitalized. Old stumps, from which the crowns have long been broken, frequently show an extraordinary growth. They seem to act as their own stimuli, and the osteoblasts, under the irritation of the mere presence of such roots, deposit upon them a layer that is out of all proportion to the original growth. Sometimes two fangs of a bicuspid or molar become united by an hypertrophic growth, the septum of true bone between them being all absorbed away. In the case of roots that have long been in an abscessed condition, it will be found that where the pericementum has been destroyed there will be no hypertrophy, but upon those parts of the root which still retain their attachment, a considerable growth will not unfrequently be seen.

The teeth which are the most subject to these growths are the molars and the bicuspids. It is somewhat singular that it is seldom or never found upon the incisors. Why this should be the case it is hard to tell, for these teeth are certainly subjected to all the irritations to which the others are liable, yet they seem exempt.

The pressure produced by these extreme growths is sometimes very great, and only for the unusual mobility of the maxillary tissues it might induce serious results. There is, primarily, a thickening of the pericemental membrane, and this will, to a certain extent, raise the root from the socket and give room for the initial deposit. The continuance of the growth will induce an absorption of the bone, and thus a cavern will be formed in the maxilla, the space being occupied by the enlarged root. Occasionally the hypertrophy involves the maxillary sinus, and serious inflammation of the lining membrane of that cavity ensues, thus complicating the case and making the determination more difficult.

The diagnosis of exostosis is never entirely clear, unless it shall have proceeded far enough to become visible upon the surface. The pain accompanying it is seldom severe, but it is usually persistent and annoying. A sense of uneasiness is constant. There is commonly an increased sensitiveness in the pulp, if it be alive, and in the case of dead roots they are sore and irritable. Yet the annoyance is not so great or so well defined as to make the patient seek the dentist for relief, and in most cases only extraction, that may be said to be accidental, reveals the condition. In a few instances the enlargement is visible from the outside, and it is then possible to proceed as in other bony tumors. We may open through the plate of the alveolus, which in such instances is very thin, and with chisels, gouges, and engine burs, remove the excrescent growth. This operation will, however, seldom be called for, owing to the difficulty of making an exact diagnosis, and the fact that it is usually teeth not worth the interference that are affected with exostosis.

When the antrum of Highmore is involved by the growth, extraction presents the only practicable remedy. But it is very hard to determine when this is the case. There is little doubt that many obscure cases of antral disease, that persist despite judicious treatment, are due to exostosed roots of teeth, which penetrate the sinus. There is little to direct the attention of the dentist to such cases, unless an extensive opening shall have first been made.

It is always well in these instances to carefully examine the maxillæ for any signs of osteophytes or osteoma, for in certain strumous diatheses exostoses are very common, and they may appear not alone upon the teeth, but along the border of the alveolus and upon the body of the bone.

In exostosed teeth the pericementum is always in an irritable condition, and this affords a slight clue in diagnosis. The tooth may appear a little elongated, or in case of a dead root it may be apparently pushed up out of its socket, but the feeling will not be as intense as in pericementitis.

Upon extraction of a tooth with an hypertrophy of the cementum, the pericemental membrane will be found in a highly vascular condition, though not so extremely congested as in pericementitis. It seems in a chronic condition of inflammation, and the thickening is at times considerable. It is this kind of low chronic inflammation, that affords us the most reliable indication in those cases which give no external sign of thickening.

There is a tumefaction of the jaw that sometimes succeeds acute periosteal or pericemental inflammation, which might possibly be mistaken for the thickening of exostosis. But in these cases there is no sensible line of demarcation. There is an apparent swelling of the tissue of the jaw, but it feels smooth and regular, with no definite edges. The swelling is also comparatively rapid in its growth, and is more apt to be tender to the touch. It is as hard and apparently as dense as the bone, but it usually disappears upon the use of the proper remedies. It is frequently the effect of acute pericementitis, but just what its character is has not been definitely determined. Garretson says that it is extra-, and not intra-maxillary, and is simply a periosteal exudate. (A system of Oral Surgery, p. 907.) But the extreme induration of such enlargements would seem to indicate a partial organization. This tumefaction is not infrequently found over the roots of teeth that have long contained large amalgam fillings. In those cases it is sometimes very persistent, but usually disappears upon the removal of the tooth. If the fillings be taken out and the pulp chambers and canals treated with some form of an iodine preparation, it will usually slowly disappear, being absorbed away.

I am not aware that any specific general medication has been recommended in cases of exostosis. The condition, though frequently the accompaniment of some cachectic state, is local in its nature, and only local surgical interference is called for in its treatment.

